# Cross-stage immunity for malaria vaccine development

**DOI:** 10.1016/j.vaccine.2015.09.098

**Published:** 2015-12-22

**Authors:** Wiebke Nahrendorf, Anja Scholzen, Robert W. Sauerwein, Jean Langhorne

**Affiliations:** a Mill Hill Laboratory, The Francis Crick Institute, London, United Kingdom; b Department of Medical Microbiology, Radboud University Medical Center, Nijmegen, The Netherlands

**Keywords:** Malaria, Vaccine, Immunization, Cross-stage, Pre-erythrocytic, Blood-stage, AMA-1, apical membrane antigen 1, CPS, chemoprophylaxis and sporozoites, iv, intravenous, MSP-1, merozoite surface protein 1

## Abstract

A vaccine against malaria is urgently needed for control and eventual eradication. Different approaches are pursued to induce either sterile immunity directed against pre-erythrocytic parasites or to mimic naturally acquired immunity by controlling blood-stage parasite densities and disease severity. Pre-erythrocytic and blood-stage malaria vaccines are often seen as opposing tactics, but it is likely that they have to be combined into a multi-stage malaria vaccine to be optimally safe and effective.

Since many antigenic targets are shared between liver- and blood-stage parasites, malaria vaccines have the potential to elicit cross-stage protection with immune mechanisms against both stages complementing and enhancing each other. Here we discuss evidence from pre-erythrocytic and blood-stage subunit and whole parasite vaccination approaches that show that protection against malaria is not necessarily stage-specific. Parasites arresting at late liver-stages especially, can induce powerful blood-stage immunity, and similarly exposure to blood-stage parasites can afford pre-erythrocytic immunity.

The incorporation of a blood-stage component into a multi-stage malaria vaccine would hence not only combat breakthrough infections in the blood should the pre-erythrocytic component fail to induce sterile protection, but would also actively enhance the pre-erythrocytic potency of this vaccine. We therefore advocate that future studies should concentrate on the identification of cross-stage protective malaria antigens, which can empower multi-stage malaria vaccine development.

## Highlights

• Antigens are shared between liver and blood-stage malaria parasites.
• Cross-stage antigens can mediate protection which is life cycle stage transcending.
• Multi-stage malaria vaccine development should identify cross-stage antigens.

## 1 Background

Malaria remains a major global health scourge and there is a general consensus that elimination and eradication efforts will not be successful without an effective malaria vaccine. In malaria endemic areas, immunity against severe disease caused by blood-stage parasites can be acquired after only one or two infections, while infections with high parasite densities still occur [1]. Vaccines targeting blood-stage parasites should equally induce control of (severe) disease but ultimately also clearance of blood-stage parasites. This is essential as gametocytes form during blood-stage infection and transmission to mosquitoes can otherwise continue.

There is only very limited evidence for protection against pre-erythrocytic malaria parasites (sporozoites and liver-stage parasites) in naturally exposed populations [2], [3], [4]. Pre-erythrocytic vaccines aim to outperform naturally acquired immunity by targeting the clinically silent stages of infection thus precluding any parasites reaching the blood stream. This would abolish any symptoms of malaria and additionally block transmission. The risk of such an approach is however that breakthrough blood-stage infections can cause severe complications, if the pre-erythrocytic vaccine is only partially effective. Therefore a blood-stage component should be included to minimize this risk [5], [6]. This is especially important since it was suggested that declining transmission intensity and thus reduced boosting of clinically protective blood-stage immunity could in fact increase overall malaria morbidity [7]. The desired scenario would therefore be to develop a multi-stage malaria vaccine that minimizes both transmission and disease [8].

## 2 Hypothesis: Shared antigenic targets between liver and blood-stage parasites can induce cross-stage immunity

Given that there are shared antigens between the different life cycle stages of the malaria parasite [9], it is possible that functional immunity to pre-erythrocytic and blood-stage parasites could enhance each other, offering an intriguing possibility for development of a multi-stage malaria vaccine. Evidence for cross-stage immunity comes from several studies [10], [11], [12], [13]. For instance, apical membrane antigen (AMA)-1 and the unprocessed form of merozoite surface protein (MSP)-1 are highly abundant in blood-stage parasites with roles in erythrocyte invasion [14], [15]; however these antigens are also expressed by sporozoites and liver-stage parasites [16], [17]. In human volunteers immunized with AMA-1 the number of blood-stage parasites during the first blood-stage cycle after mosquito bite challenge infection was about 7-times lower compared to non-immunized controls, suggesting that pre-erythrocytic immune responses may have eliminated sporozoites or infected hepatocytes [13]. Indeed an 80% reduction of liver-stage parasite burden following sporozoite challenge was shown in mice immunized with AMA-1 [10]. After vaccination of humans with AMA-1 and MSP-1, time to diagnosis, which was delayed in the vaccinees, significantly correlated with liver-to-blood parasite levels but not blood-stage multiplication rates. This suggests again that this vaccine may induce pre-erythrocytic rather than the originally intended blood-stage immunity [12]. These examples indicate that only one or two malarial antigens, which are expressed in both liver- and blood-stage parasites, can induce cross-stage protective immunity. Whole parasite vaccines that allow exposure to many parasite antigens should therefore provide greater potential for cross-stage immunity, which would enhance protection induced by pre-erythrocytic and blood-stage parasites. We therefore discuss evidence for cross-stage immunity from different whole parasite vaccination approaches, which offers the opportunity to identify as yet unknown cross-protective antigens for multi-stage malaria vaccine development.

## 3 Evidence for cross-stage immunity from pre-erythrocytic whole parasite vaccination approaches

The proteome of liver-stage parasites becomes increasingly similar to blood-stage parasites as liver development proceeds [9]. Late liver-stage schizonts contain up to 40,000 merozoites each [18], which can upon release invade erythrocytes. Furthermore the amount of parasite antigen increases as the parasite matures in hepatocytes. Killed sporozoites that fail to invade hepatocytes are incapable of inducing protection [19], [20], suggesting that liver-stage development is indispensable for induction of protective immunity. Irradiation of sporozoites, which arrests their development early during the liver-stage [21], [22], induces immunity to pre-erythrocytic stages only [19], [23]. A very limited number of studies have, however, investigated whether there are significant immune responses, or any level of protection against blood-stage parasites. One report from Krzych et al. [24] suggests a more in-depth study of cross-stage immune responses induced by irradiation attenuated sporozoites might be valuable, as T cells from human volunteers immunized with irradiated sporozoites responded to both pre-erythrocytic and blood-stage antigens, including MSP-1. This response was greater in immunized volunteers, who were protected from challenge infection, than in unprotected volunteers and comparable to malaria-experienced individuals [24]. Therefore, immunization of humans with irradiated sporozoites leads to the induction of immune responses recognizing blood-stage antigens. CD8T cells, which were shown to be essential for pre-erythrocytic protection following irradiated sporozoites immunization [25], proliferate more strongly in mice if in addition to *Plasmodium berghei* irradiated sporozoites they were exposed to blood-stage parasites [26]. This suggests that blood-stage infection can enhance pre-erythrocytic vaccine efficacy. Furthermore in a *P. berghei* infection model in mice, multiple booster immunizations with high numbers of irradiated sporozoites resulted in delayed patency and reduced peak blood-stage parasitemia after sporozoite challenge (Nganou-Makamdop K, personal communication), suggesting that cross-stage protective responses targeting blood-stage parasites may have developed. It has however also been observed that a fulminant blood-stage infection can suppress protective immune responses elicited by irradiated sporozoites against liver-stage antigens [27]. Possible negative interferences between immunity directed against liver and blood-stage parasites can therefore not be excluded.

Targeted deletion of parasite genes important for liver-stage development is an alternative strategy to arrest parasite development in hepatocytes. Similar to irradiated and chemically attenuated [28] sporozoites, immunization with knock-out parasites that arrest during the early liver-stage (e.g. *uis* 3 [29], *uis* 4 [30], *p36p* [31], *sap-1* [32], *p52*/*p36* [33], [34]) results in pre-erythrocytic immunity. Late arrest during liver-stage development [35], however, appears to increase the chance of cross-stage immunity: Immunization with *P. yoelii fabb/f* knockout sporozoites can control and clear blood-stage parasitemia following challenge with blood-stage parasites, possibly by inducing an effective T cell response [36]. This is the first direct evidence that immunization with an attenuated parasite, which does not develop beyond liver-stage, can elicit blood-stage immunity.

**Fig. 1:**
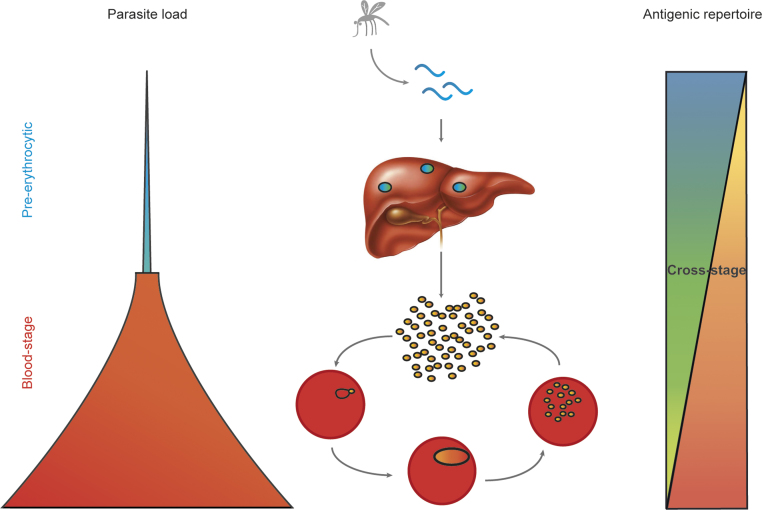
Shared antigens between liver and blood-stage malaria parasites can induce cross-stage immunity. Malaria infection is initiated by an infectious mosquito bite, which inoculates a few sporozoites into the skin. Sporozoites then migrate to the liver and invade hepatocytes. During pre-erythrocytic development (which last 7 days for *Plasmodium falciparum*) parasite load slowly increases as *Plasmodium* matures in the liver. In addition, the antigenic repertoire becomes increasingly similar to blood-stage parasites [9] and cross-stage antigens are expressed. Late liver schizonts contain up to 40,000 merozoites [18], which are released into the blood-stream as merosomes surrounded by a host cell membrane [53]. When each merozoite invades an individual red blood cell parasite load rises rapidly. Blood-stage parasites mature from rings to trophozoites and schizonts and parasite load increases exponentially with each new replication cycle (*P. falciparum*takes 48h to complete one blood-stage cycle). Apart from typical blood-stage antigens infected erythrocytes also express cross-stage antigens, which are shared with pre-erythrocytic parasites. There is evidence from subunit and whole parasite immunization approaches that shared antigens between pre-erythrocytic and blood-stage parasites could induce cross-stage immunity. Characterization of these antigens would greatly facilitate multi-stage malaria vaccine development.

Cross-stage immunity therefore appears to be more efficient if liver-stage parasites arrest late in development as their antigenic profile becomes similar to blood-stage parasites [9] and the amount of antigen increases (Fig. 1). Hence antigens expressed in late liver-stage parasites are, under certain conditions, capable of mediating not only pre-erythrocytic protection, but also reduce the risk of blood-stage breakthrough infection by inducing effective blood-stage immunity.

## 4 Evidence for cross-stage immunity from whole blood-stage parasite vaccination approaches

*Plasmodium* replicates massively in the liver such that one infected hepatocyte can release up to up to 40,000 blood-stage parasites [37]. Also due to their subsequent exponential multiplication, blood-stage parasites are hence much more numerous than pre-erythrocytic parasites, which increases their potential to present protective antigens successfully. The possibility that immune responses against these antigens might not be only specific for blood-stage parasites, but could also target pre-erythrocytic stages has however hardly been investigated (Fig. 1).

Disruption of the *purine nucleoside phoshphorylase* gene (*pnp*) [38] or *nucleoside transporter 1* (*nt1*) gene [39] in *P. yoelii* gives rise to severely attenuated blood-stage infections, and mice that had undergone an infection with these knockout parasites did not develop detectable patent blood-stage parasitemia after infectious mosquito bite or sporozoite challenge [38], [39]. This could represent effective blood-stage or pre-erythrocytic immunity, since a reduction in liver parasite burden, which is the only direct evidence for pre-erythrocytic protection, was not shown. Direct evidence for pre-erythrocytic protection elicited by blood-stage parasites comes from mice that received a prophylactic treatment with chloroquine and were simultaneously infected with *P. yoelii* blood-stage parasites. Liver parasite burden is significantly reduced in these mice following sporozoite challenge [40].

It is presumed that the main purpose of blood-stage components in a multi-stage vaccine is to protect against breakthrough blood-stage infection [41], if the pre-erythrocytic components are only partially effective. However, since whole parasite blood-stage immunizations have the potential to also induce cross-stage protective responses enhancing immunity against pre-erythrocytic stages, blood-stage antigens might be even more valuable for multi-stage malaria vaccine development.

## 5 Is cross-stage immunity responsible for the unprecedented efficiency of chemoprophylaxis with sporozoites immunization?

The induction of cross-stage immunity should be facilitated by exposure to both pre-erythrocytic and blood-stage parasites during immunization. Chemoprophylaxis with sporozoites (CPS) immunization, which uses infectious wild-type sporozoites combined with prophylactic antimalarial drug treatment (often chloroquine), allows the immune system to experience all vertebrate *Plasmodium* life cycle stages including sporozoites, infected hepatocytes and blood-stage parasites. CPS immunization was first described in rodents [42], [43] and was later shown to induce long lasting sterile protection against homologous mosquito-bite challenge in human volunteers [44], [45], [46]. In humans CPS immunization is about 20 times more efficient than immunization with irradiated sporozoites [23], [47]. We therefore hypothesize that the completion of liver-stage development and the exposure to blood-stage parasites [40] during CPS immunization may enhance the protective efficacy by inducing protective cross-stage responses. CPS immunization using primaquine – a drug that primarily targets liver-stage parasites – substantially reduces the number of sterilely protected mice thereby strengthening this hypothesis [20]. In further support of this, mice that have experienced a self-cured infection with *P. chabaudi* blood-stage parasites derived from a donor mouse infected by mosquito bite have a substantially reduced liver-parasite burden following mosquito bite challenge [48]. Cross-stage immunity elicited by blood-stage parasites may hence contribute to the unprecedented efficiency of CPS immunization to induce sterile pre-erythrocytic protection in human volunteers [44], [45], [46]. Importantly exposure to blood-stage parasites during CPS immunization may significantly contribute to the observed pre-erythrocytic protection against mosquito bite challenge, but does not appear to protect against direct blood-challenge within the first four cycles of blood-stage replication [45]. In human volunteers CPS immunization induces both T cell and antibody responses, which may be implicated in protection [46], [49], [50], [51]. Protective target antigens and the exact mechanism of protective immunity are however still unclear.

## 6 Conclusion: Importance of studying cross-stage immunity for malaria vaccine development

Cross-stage immunity would be a powerful means to improve protective efficacy of malaria vaccines, however data to support this hypothesis are sparse. During liver-stage development both the amount of antigen and the antigenic similarity to blood-stage parasites increases (Fig. 1). Thus as *Plasmodium* matures in the liver, it is potentially capable of inducing immunity not only against pre-erythrocytic but also against blood-stage parasites [36]. Equally, exposure to attenuated blood-stage parasites could protect against sporozoite challenge [40].

Future studies should therefore investigate the potential of cross-stage immunity directly. In our opinion whole parasite immunization approaches are particularly useful to answer the question whether cross-stage immunity contributes to protection and to identify protective cross-stage antigens for subunit malaria vaccine development. If however a whole parasite immunization strategy were proven to be safe, effective and practical first, there would be no reason not to implement it. Pre-erythrocytic vaccines should evaluate blood-stage protection after direct injection of blood-stage parasites, while blood-stage vaccines should demonstrate whether liver-parasite burden is reduced following sporozoite challenge. If cross-stage immune responses can be elicited, target antigens and crucial immunological mechanisms mediating it should be characterized using proteome-wide screening approaches (immunomics) of antibody and T cell reactivity comparing protected and unprotected individuals [51], [52]. Selecting antigens capable of inducing cross-stage protection could greatly facilitate the development of a multi-stage malaria vaccine by increasing potency.

## Conflict of interest statement

The authors declare no conflict of interest.
